# Advancements in the application of nanotechnology for the management of epileptic seizures

**DOI:** 10.1186/s42494-024-00171-6

**Published:** 2024-07-23

**Authors:** Honglu Ping, Ding Ding, Guoxing Zhu, Jianhong Wang, Jun Zhang

**Affiliations:** 1 Department of Neurology, Huashan Hospital, Fudan University, 12 Wulumuqi Middle Road, Shanghai, 200040 China; 2 Department of Radiology, Huashan Hospital, State Key Laboratory of Medical Neurobiology, National Center for Neurological Disorders, Fudan University, Wulumuqi Middle Road No.12, Shanghai, 200040 China

**Keywords:** Epilepsy, Nanomaterials, Stimulus response system, Blood-brain barrier, Drug delivery

## Abstract

Epilepsy is a common yet complex neurological disorder. Historically, antiseizure medications (ASMs) have faced challenges in crossing the blood-brain barrier (BBB) and targeting the epileptogenic zone, creating a bottleneck in seizure management. Certain nanomaterials can facilitate drug penetration through the BBB and enable stimulus-responsive drug release, thereby enhancing targeted and efficient drug utilization while reducing adverse reactions in other brain tissues and peripherally. This article reviews the current researches on stimulus-responsive nanosystems applicable in antiepileptic therapy, as well as nanotechnology applications that improve the brain delivery of ASMs.

## Background

Epilepsy is one of the most common brain disorders, affecting approximately 51 million people worldwide [1]. Epidemiological data shows that there are about 10 million epilepsy patients in China [2]. Despite reasonable pharmacological treatment, about 30% of these patients do not respond to any form of antiseizure medications (ASMs), known as drug-resistant epilepsy (DRE) [1]. Uncontrollable epileptic seizures severely harm the physical and mental health of patients, significantly increasing mortality rates [3]. DRE is associated with increased risks of mortality due to various complications, including status epilepticus, trauma, sudden unexpected death in epilepsy, and aspiration [4]. Furthermore, these patients are more susceptible to psychological disorders [5–7], a consequence of both the stigma surrounding epilepsy and the adverse effects of long-term use of multi drug combination [8, 9]. The pathophysiological mechanisms underlying DRE remain inadequately understood. However, recent research has put forth multiple hypotheses to explain its etiology [10]. The central transporter hypothesis [11], for instance, has suggested that drug resistance in DRE may be due to the overexpression of multidrug transporters on the blood-brain barrier (BBB), which limits the penetration of ASMs. Increasing the dosage of medication to enhance the concentration of ASMs in the brain can exacerbate the incidence of adverse reactions, thus affecting the quality of life and even harming the physical health of patients. ASMs not only reduce the excitability of neurons in the epileptogenic zone but also decrease the excitability of neurons in healthy areas. This often leading to various side effects and adverse reactions such as drowsiness, headache, dizziness, ataxia, nausea, vomiting, and loss of appetite [12], significantly affecting the daily life quality of epilepsy patients [13]. Therefore, maintaining an effective concentration of ASMs in the brain without increasing the dosage, and further achieving targeted drug delivery, is of great significance for the precision restraint of epilepsy. Addressing these challenges, recent advancements in nanotechnology have shown promise. Novel nano-carriers have been developed to enhance the delivery of ASMs across the BBB, improving drug efficacy and utilization. Additionally, nano-responsive systems offer more precise drug release, enabling targeted therapy and potentially reducing adverse reactions. This study provides an overview of these nanotechnological advancements in enhancing BBB permeability and responsive drug delivery for the control of seizures in epilepsy.

## Nanotechnology in enhancing ASMs delivery across the BBB

**Fig. 1:**
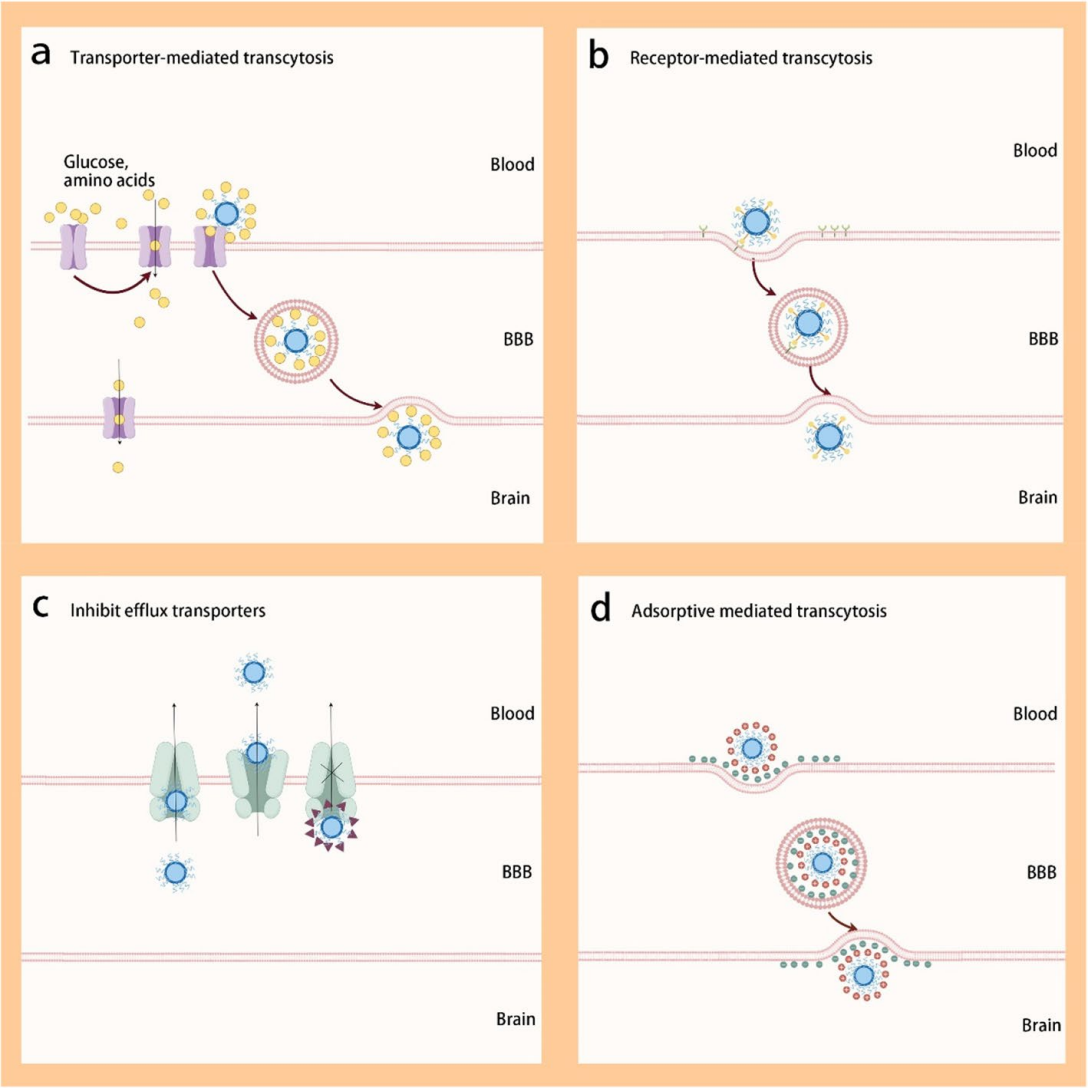
Intracerebral drug delivery strategy utilizing nanoparticles targeting endogenous points across the BBB. This includes (**a**) Transporter-mediated transcytosis, (**b**) Receptor-mediated transcytosis, (**c**) Inhibit efflux transporters, and (**d**) Adsorptive mediated transcytosis

The BBB is a highly selective permeability barrier formed by the endothelial cells of cerebral microvessels and neuroglial cells. It rigorously regulates the exchange of substances between the blood and brain tissue, thereby maintaining cerebral homeostasis and protecting neural tissue from potentially harmful exogenous substances [14]. Due to its high biological selectivity, it is difficult for ASMs to cross the BBB. To augment the cerebral delivery of ASMs, advanced strategies in nanocarrier design are employed. These include surface modification with substrates of BBB-specific transport proteins, ligand-receptor targeting using specific receptor ligands, incorporation of cell-penetrating peptides, and the inhibition of efflux transporters (Fig. 1). Such approaches enhance transcytosis-mediated drug penetration across the BBB, thereby optimizing the drug's locational specificity and bioavailability within the brain, and significantly increasing its therapeutic concentration in the cerebral milieu.

### Targeting brain transport proteins for enhanced BBB drug delivery

Vital cerebral nutrients, such as glucose and amino acids, traverse the BBB via a carrier-mediated transport mechanism. This intricate process necessitates the involvement of specific transport proteins, whose substrates can be ingeniously exploited as ligands in the realm of nanotechnology-enhanced drug delivery. A quintessential example is the glucose transporter 1 (GLUT1), where glucose, its natural substrate, plays a pivotal role. Ingenious modification of nanocarrier systems with glucose or its analogs can significantly optimize the cerebral distribution of therapeutic agents, offering a promising avenue for enhanced drug delivery to the brain. Zhao et al. [15] encapsulated carbamazepine within the lipophilic bilayer of 1,2-distearoyl-sn-glycero-3-phosphoethanolamine (DSPE), exposing hydrophilic glucose on the surface to create a nanocarrier drug delivery system. The results indicated that this modified carbamazepine liposome exhibited a higher release rate and was successfully internalized into neurons. Similarly, Yilmaz et al. [16] coated the ASM lacosamide with glucose, achieving comparable outcomes. Zhou et al. [17] targeted GLUT1 using a glucose analogue, de-hydroascorbic acid, which also enhanced the effective concentration of the medication. Therefore, glycosylation of the nanosystem's surface can facilitate the more effective passage of drugs through the BBB.

Similarly, L-type amino acid transporter 1 (LAT1) is a protein in the brain responsible for the transport of amino acids, with tryptophan being one of its exogenous substrates. During epileptic seizures, due to the substantial consumption of tryptophan, the BBB becomes more sensitive to it. Exploiting this characteristic, Wang et al. [18] covalently attached non-radioactive alpha-methyl-L-tryptophan to magnetic nanoparticles, enabling their uptake by epileptic brain tissue and passage through the BBB, thereby aiding in the localization of epileptogenic foci. Lamotrigine (LTG), a first-line ASM, is readily metabolized in the liver. Liu et al. [19] encapsulated LTG with Pluronic P123/F127 mixed micelles and targeted LATI using a tryptophan derivative (TD).The results demonstrated that these TD-functionalized nanoparticles could deliver the drug more effectively to the brain, particularly to the hippocampus, thereby enhancing the bioavailability of LTG.

### Utilizing receptor-mediated transcytosis for enhanced BBB drug delivery

The surface of the BBB possesses receptors that mediate transcytosis [20]. By conjugating their ligands to the exterior of nanosystems, it is possible to achieve specific binding to these receptors, thereby facilitating passage through the BBB. For instance, angiopep-2, a ligand for low-density lipoprotein receptor-related protein 1 (LRP1), has been chosen by many researchers as a targeting moiety for drug delivery to enhance the cerebral concentration of ASMs, details to follow later [21, 22]. Similarly, transferrin, a ligand for the transferrin receptor (TfR), targeting the BBB, has been widely used for enabling nanosystems to cross the BBB [23–28]. However, under physiological conditions, TFR is often saturated with endogenous ligands, leading to suboptimal targeting [29]. Therefore, the use of monoclonal antibodies against TFR has been proposed to induce endocytosis in the absence of competition from endogenous ligands. Hou et al. [30] used the TfR monoclonal antibody D-form T7 (D-T7) peptide to increase LTG concentration in the brain. LTG was encapsulated within a core of poly lactic-co-glycolic acid (PLGA), covered by a lipid shell, and combined with D-T7 peptide (targeting the BBB) and Tet1 peptide (targeting neurons) to form a nanosystem. Both in vitro and in vivo experiments demonstrated that this nanosystem had excellent neuron targeting, antiseizure, and protective effects.

In recent years, Li et al. [31] have utilized phage display technology to identify a peptide with effective brain-targeting properties, named PepTGN. Phenytoin (PHT) sodium, a classic antiseizure medication, has a narrow therapeutic window and low BBB permeability, leading to intolerable side effects and resistance. Zhao et al. [32] developed a hepatitis B core protein nanocage, into which the PepTGN was inserted, enhancing the delivery of PHT to the brain. The results indicated that in a pilocarpine-induced epilepsy model, this nanocage effectively targeted brain tissue, significantly improving the antiseizure efficacy of PHT.

### Utilizing adsorptive mediated transcytosis (AMT) for enhanced BBB drug delivery

Nanomaterials can also traverse the BBB via AMT [33]. The external and internal surfaces of brain endothelial cells carry a negative charge, and when positively charged substances come into contact with the cell membrane, electrostatic interactions are triggered. Therapeutically, AMT can be achieved by constructing cationic surface charges on nanoparticles or by coupling nanoparticles with positively charged substances [34], such as cell-penetrating peptides. The trans-activator of transcription (TAT) peptide, derived from the human immunodeficiency virus, is a well-known cell-penetrating peptide. TAT peptides can penetrate cell membranes and transport various molecules such as proteins, nucleic acids, and nanoparticles into cells [35, 36]. Hou et al. [37] created a brain-targeted nanosystem for controlling epileptic seizures by coupling different peptides to the shell lipid of a core composed of PLGA and LTG, and evaluated and compared the performance of six peptides (T7, D-T7, GSH, TGN, CGN, and TAT). In vitro experiments showed that nanoparticles modified with TAT had the highest internalization effect on mouse brain microvascular endothelial cells (a BBB model) and hippocampal neurons. Another example of applying TAT involves incorporating PHT into calcium phosphate (CaP) nanoparticles through biomineralization, followed by surface modification with the PEGylated TAT peptide. Experiments demonstrated that CaP@PHT-PEG-TAT could effectively deposit in the mouse brain without causing significant collateral toxicity. Once absorbed by cells, the nanosystem releases Ca^2+^ and PHT into the cytoplasm in the acidic environment of lysosomes [38].

### Inhibiting efflux transporters for increased brain drug concentration

Due to the presence of efflux transporters, drugs that enter the BBB may be expelled by the efflux system, preventing them from exerting their therapeutic effects. This is also a contributing factor to the development of DRE [39]. Among these, P-glycoprotein (P-gp), also known as multidrug resistance protein 1, is a representative efflux transporters responsible for expelling various ASMs [40]. Amphiphilic substances such as d-alpha-tocopheryl polyethylene glycol succinate (TPGS), poloxamer, and polysorbate 80 have been used to increase drug concentrations in the blood, thereby countering the efflux action of P-gp.

Lombardo [41] first observed that carbamazepine, phenobarbital, and PHT, through their interaction with the pregnane X receptor (PXR), induce the production of P-gp and other transport proteins in rat brain endothelial cell lines. PXR is a key regulatory factor in the expression of transport proteins under exogenous exposure. With the induction of efflux transporters, the binding of exogenous drugs to PXR co-upregulates the drug-metabolizing enzyme cytochrome P450 3A4 (CYP3A4). Overexpression of CYP3A4 can reduce the concentration of ASMs in plasma and epileptic foci to a functionally deficient level. Thus, inhibiting PXR helps to downregulate the expression of CYP3A4, thereby increasing the plasma concentration of ASMs. Ketoconazole (KCZ), an antifungal drug, is a recognized effective PXR antagonist. However, the therapeutic concentration of KCZ is not sufficiently high or sustained to counteract the activation of PXR in the brain. Based on this, Wang et al. [42] developed a functionalized PEG-PLA nanoparticle system of KCZ to overcome the overactivity of PXR and treat drug-resistant epilepsy. Compared to monotherapy with carbamazepine, intravenous injection of NPs/KCZ and carbamazepine significantly improved spontaneous seizures in KA-induced epileptic mice. NPs/KCZ also reduced the mortality rate in mice compared to KCZ alone.

Considering that P-gp not only transports drugs but also plays a crucial protective role in limiting the entry of various harmful substances into cells, the safety concerns associated with excessive inhibition of efflux transporters must be given serious attention [43].

Beyond its therapeutic role, P-gp can also serve as a target for detecting epileptic foci in the brain. For instance, Yu et al. [44] developed a nanoparticle by coupling superparamagnetic iron oxide nanoparticles with a near-infrared probe and the targeting component pepstatin (a peptide with specific affinity for P-gp). This nanoparticle enables non-invasive quantification of P-gp expression levels in vivo. In a rat model of epilepsy, these nanoparticles were detected accumulating easily and selectively in epileptogenic brain regions through magnetic resonance and optical imaging. This P-gp-targeted nanomedicine is useful for molecular imaging of P-gp expression changes in epileptic lesions, for understanding the mechanisms of P-gp dysregulation, and for predicting intractable epilepsy.

## Application of nanomaterials in responsive release of ASMs

Traditional ASMs have low targeting specificity, and controlling the precise release of these drugs in epileptogenic areas has always been a key area of scientific endeavor. Based on different response mechanisms, nanosystems developed to aid the delivery of ASMs can be categorized into electro-responsive, thermo-responsive, ROS-responsive, and pH-responsive types.

### Electro-responsive nanosystems

Based on the characteristic of abnormally high-frequency neuronal discharges during epileptic seizures, electrically-stimulated responsive drug delivery systems can be developed. Conducting polymers (CPs) are a unique class of polymeric materials that contain ionizable groups within their structure, endowing them with the unique ability to change shape and size upon electrical activation [45]. Examples include materials like polyaniline, polypyrrole, polythiophene, polypyrrole, and polyethylene. However, when used alone, CPs often have low solubility, insufficient drug loading capacity, and poor stability. Therefore, CPs are commonly incorporated into various nanostructures or doped with additives, such as nanogels and self-assembling micelles, to overcome these limitations.

For instance, electric field-responsive nanogels exemplify a "win–win" situation by combining the advantages of both CPs and hydrogels, including ample drug loading space, excellent biocompatibility, and electrochemical activity [46]. Hydrogels contain a large number of hydrophilic groups that absorb water, forming a stable three-dimensional network structure. This highly porous structure allows drugs to be loaded into the gel matrix, serving as a drug reservoir. Additionally, electrically-responsive control of drug release minimizes peripheral adverse reactions. For example, PHT is a traditional ASM known for its effectiveness in treating focal epileptic seizures [47], but it has been excluded from first-line epilepsy treatments due to its peripheral side effects [48]. Reducing peripheral adverse reactions while increasing cerebral drug concentration remains a challenge. Wang et al. [21] used 2-(dimethylamino)ethyl methacrylate (hydrophilic group of the hydrogel), styrene (forming the internal hydrophobic area of the hydrogel), and sodium 4-vinylbenzene sulfonate (NaSS) as monomers (acting as CPs for electrical responsiveness), with N, N'-methylene bisacrylamide as the crosslinker, and potassium persulfate-sodium metabisulfite as the redox initiator. They modified the nanogel system with the brain-targeting peptide angiopep-2, creating an electrically-responsive nanogel system for delivering PHT. In the nanogel, the sulfonic acid groups of NaSS, under the influence of an electric field, cause rapid swelling of the gel due to electrostatic repulsion between charges, increasing the particle size and thereby releasing PHT. This increases the peak concentration of PHT in the brain, significantly enhancing the total amount and duration of PHT retention in the brain, and providing more effective antiseizure activity with an extended period of seizure control.

**Fig. 2:**
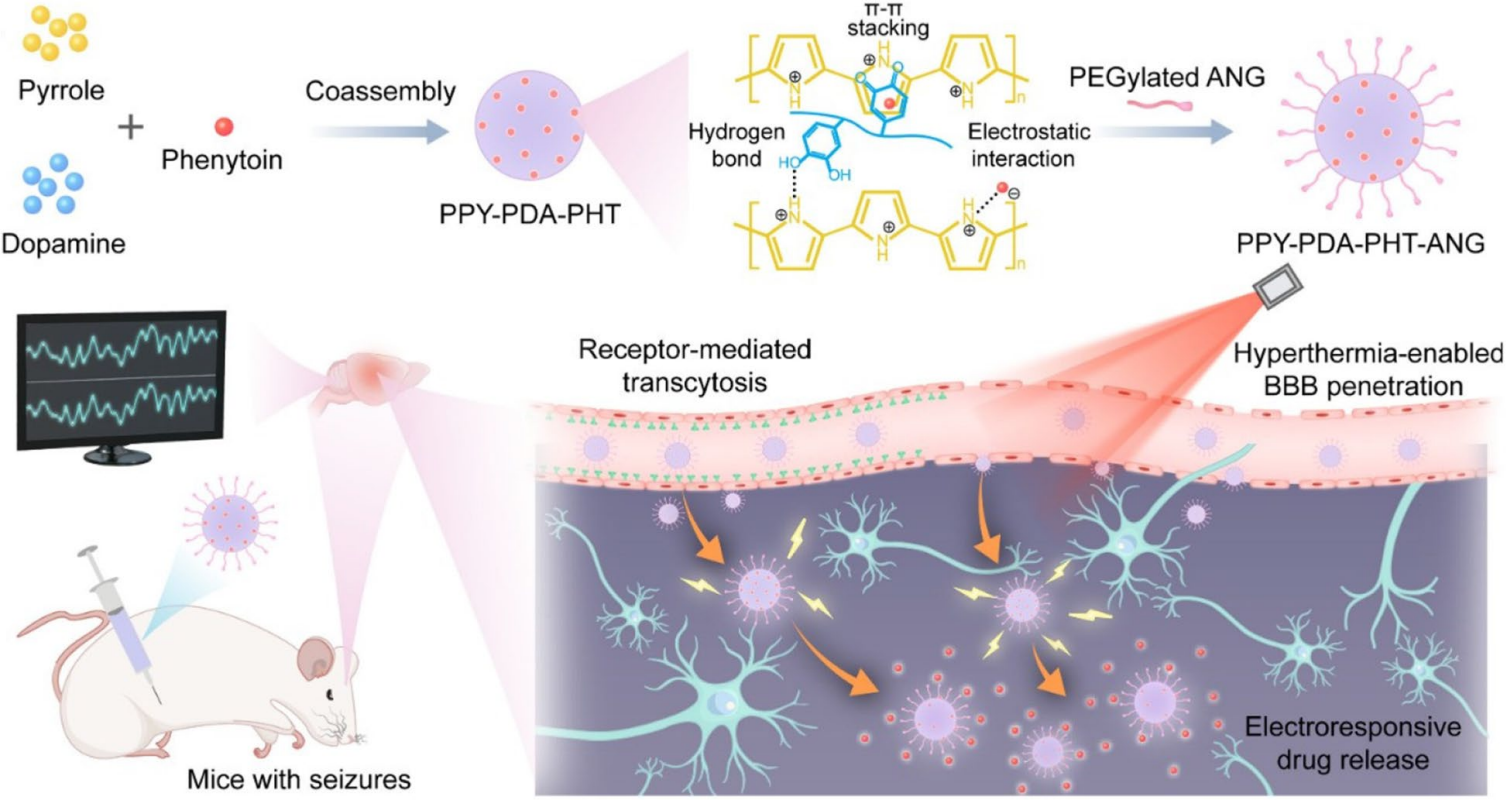
Schematic illustration of the synthesis of electroresponsive brain-targeting nanoparticles using PPY and PDA. Permission was granted by Wu D et al. (©American Association for the Advancement of Science [22]) to reuse this figure

Some researchers have also utilized CPs to form self-assembling micelles for the purpose of achieving electrically-responsive drug delivery. Micelles are colloidal carriers formed by the self-assembly of amphiphilic molecules. Wu et al. [22] used polyvinyl alcohol as a stabilizer and ammonium persulfate as an oxidant to load the drug PHT into polymer nanoparticles along with polypyrrole (PPY) and polydopamine (PDA) (Fig. 2). PDA endows the nanomicelles with excellent hydrophilicity, increasing the water solubility of the conductive material PPY, and enhancing the sensitivity of the nanoparticles to electrical stimulation within a certain range (peaking at a dopamine mass ratio of 5%), allowing for rapid release of ASM during epileptic discharges. He also noted the unique absorbance of PDA in the second near-infrared window (1000 to 1700 nm). The heat generated by photothermal conversion can increase the permeability of the BBB, enhancing the non-invasive delivery of drugs to deeper brain tissues. The surface functionalization with the brain-targeting peptide angiopep-2 and radiation-triggered hyperthermia synergistically promote drug delivery in the brain. An electric field of 50μA is sufficient to trigger drug release, and the PPY-PDA-PHT nanoparticles can respond to intermittent discharges for sustained drug release. Under "on–off" cycles of electrical stimulation, the drug is released in the "on" state, while leakage in the "off" state is negligible. This represents a successful attempt to reduce peripheral adverse reactions of PHT medication.

**Fig. 3:**
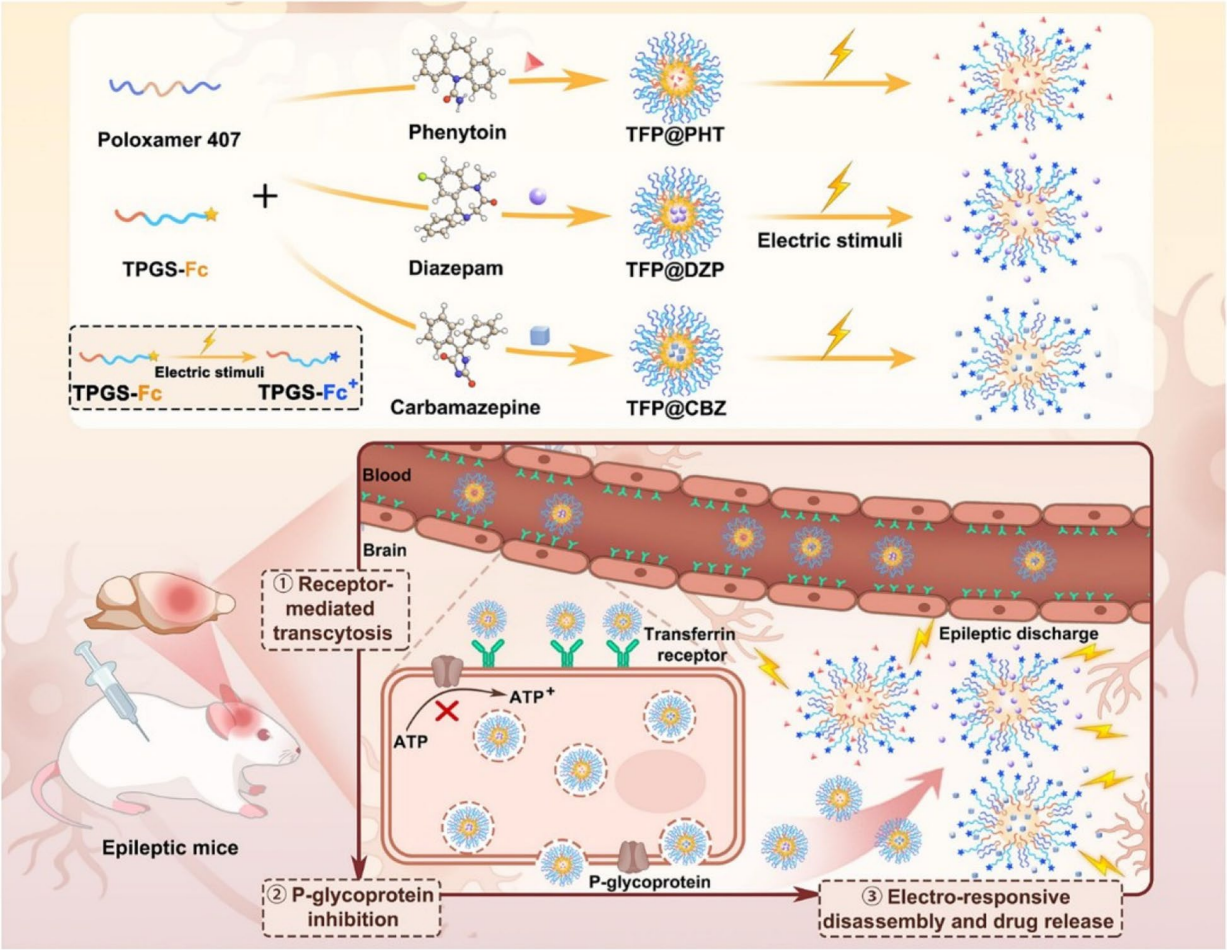
Schematic diagram of Fc electrical response mechanism. Permission was granted by Zhang Q et al. (©Elsevier [50]) to reuse this figure

Ferrocene (Fc) is also a common electro-responsive group. In its reduced state, Fc is hydrophobic and can load hydrophobic drug molecules. By oxidizing Fc to make it hydrophilic, drugs can be released. Wang [49] proposed an electro-responsive MRI contrast agent. Utilizing Fc as an electro-responsive group, which can be reversibly oxidized to the positively charged Fc^+^ upon electrochemical stimulation, leading to the disassembly of the polymer, it is composed of a paramagnetic polymer coating encapsulating ultrasmall superparamagnetic iron oxide (USPIO). In a mouse model of drug-resistant focal epilepsy, changes in the brain's electrical field during seizures trigger the disintegration of the contrast agent, restoring the T1-weighted MRI signal and increasing the probability of localizing epileptic foci. Zhang et al. [50] reported an electro-responsive drug delivery system based on Fc, which was prepared by coupling ferrocene carboxylic acid with TPGS and assembling with poloxamer 407 (PF127) (Fig. 3). Poloxamer is a polyethylene oxide-polypropylene oxide-polyethylene oxide (PEO-PPO-PEO) block copolymer, featuring a hydrophobic core and a hydrophilic shell, making it amphiphilic. This new nanosystem can deliver various ASMs. TPGS, an amphiphilic polymer, can penetrate the BBB through receptor-mediated endocytosis. By attaching the lipophilic end to Fc to synthesize TPGS-Fc, an electro-responsive brain-targeting nanomicelle drug delivery system is prepared. PF127 can reduce the critical micelle concentration of the nanomicelle drug delivery system, maintaining the stability of the nanosystem at low concentrations in vivo.

In summary, electro-responsive nanosystems utilize ionizable groups present in electroactive polymers such as polyaniline, PPY, Fc, and NaSS. Through electrical stimulation, these systems undergo conformational changes in volume, size, and shape. They can respond to abnormal neuronal signals during epileptic seizures, enabling “on-demand” supply of ASMs.

### Thermo-responsive nanosystems

The physical properties of drug delivery carriers that change with temperature are known as thermoresponsive systems or thermo-responsive systems. Their functionality relies on carriers responsive to heat and thermal stimuli. Thermoresponsive materials aggregate below a lower critical solution temperature and swell above this range due to decreased hydrophilicity, thereby releasing drugs [51]. The heat source can be body temperature or external stimuli, such as magnetic fields and light.

Magnetic fields are commonly used as an external thermal stimulus, with superparamagnetic iron oxide nanoparticles (SPIONs) serving as the magnetic-responsive heat source. SPIONs are a type of superparamagnetic material composed of two unpaired electrons, exhibiting unique superparamagnetism. They generate heat in an alternating magnetic field, becoming a heat source for thermo-responsive systems. For example, Huang [52] designed a magnetic-responsive, thermally-activated nanocarrier. The structure of PF127 exhibits good thermoresponsiveness, showing structural instability as the temperature drops below its lower critical solution temperature. Polyvinyl alcohol is used as a stabilizer to provide hydrogen bonds that react with PF127, forming an ultra-thin nanoshell to stabilize the carrier and prevent drug leakage. The ASM ethosuximide is encapsulated inside the nanocarrier with SPIONs at its core. Under the influence of an external high-frequency magnetic field, SPIONs generate heat, causing significant volume changes in the thermosensitive nanocarrier and triggering rapid drug release within a short period. In vivo experiments have demonstrated its capability for responsive release and immediate suppression of epileptic seizures.

### ROS-responsive nanosystems

During an epileptic seizure, the heightened excitability of the brain leads to excess production of free radicals and reactive oxygen species (ROS) in neuronal mitochondria. When the mitochondrial antioxidant system and repair processes are overwhelmed, there is an increase in steady-state ROS levels, resulting in oxidative stress damage to neurons [53]. Furthermore, oxidative stress-induced neuronal damage, along with mitochondrial dysfunction and microenvironmental changes, can exacerbate epileptic seizures, creating a vicious cycle [54].

Researchers have speculated whether ROS could be used as a potential target to trigger the release of ASMs at specific sites. Zhou [17] proposed using ROS-sensitive phenylboronic ester coupled with PEG to form an amphiphilic copolymer. ROS stimulation can transform the hydrophobicity of polymers modified with arylboronic ester side chains into hydrophilicity, thereby triggering degradation of the drug delivery system and releasing encapsulated drugs. LTG is encapsulated in micelles through self-assembly. The micelles can alter the distribution of LTG in the body, thus enhancing efficacy. This system can simultaneously inhibit abnormal discharges, reduce oxidative stress, and regulate the inflammatory microenvironment of brain foci, making it suitable for epilepsy treatment. The ROS detection system provides a targeted and dose-dependent intracellular nanodelivery system through disulfide-thiol exchange reactions. Upon oxidation by ROS, drug release is triggered, enhancing the targeting capability of the ASMs [55].

### pH-responsive nanosystems

Ordinary ASMs typically require multiple daily doses, often leading to missed doses. Missed doses of epilepsy medication can cause significant fluctuations in blood drug levels and may trigger seizures [56]. pH-sensitive nanogels can be used to create extended-release formulations of ASMs, allowing for slow release in the gastrointestinal tract [57], thereby improving patient compliance. They can also be used for targeted drug delivery in epilepsy. During epileptic seizures, due to hypoxia, ion gradients, and the presence of HCO_3_^− ^[58–63], as well as the metabolic production of CO_2_ [64] and lactate, the intracellular environment of neurons in the seizure focus becomes acidic [65], while the extracellular environment shows biphasic changes. Initially, it becomes more alkaline at the start of a seizure, then gradually more acidic over time, with longer seizures leading to more pronounced acidification [66, 67]. Between seizures, the focus area tends to be more alkaline [68], with pH values in the focus area fluctuating between 6.8 and 7.8 during and between seizures [68–71]. One study also showed that neurons acidify during seizures, while adjacent astrocytes become more alkaline [72]. Therefore, pH-responsive nanocarrier systems, which can identify the microenvironment of epileptic foci, have broad application prospects. Trivedi et al. [73] synthesized a pH-responsive polymer by polymerizing chitosan with PDMEAMA, encapsulating the glutaminase inhibitor 6-diazo-5-oxo-L-norleucine (DON). The coated pH-responsive polymer shows good sensitivity to acidic conditions, releasing the drug at pH 6.4 and resisting release at a higher pH of 7.2. Compared to DON alone, this nanosystem reduced the mortality rate in PTZ-induced mice. However, there are still many issues, such as the fact that the pH in the brain during epileptic states may not drop to 6.4 [74], and the acidic environment itself can inhibit seizures [59, 75]. Is it feasible to wait until the brain's pH drops to an acidic environment to release the drug for seizure control? And in terms of the control effect of the nanosystem in mice, compared to DON alone, the duration of seizures and the time to return to normal were not reduced. Therefore, further exploration is needed on how pH-responsive nanosystems can effectively control epilepsy.

Polymers with weak acidic or basic residues that exhibit unique properties at different pH levels are known as pH-responsive polymers. At lower pH values, these weak acidic or basic side groups accept protons, while at higher pH values, they release protons. Therefore, based on their pKa values, these polymers exhibit polyelectrolyte characteristics in acidic or alkaline environments. This transition from non-ionic to ionic alters the degree of hydrophilicity, causing the polymer chains to precipitate or dissolve, and their hydrogels to swell or contract, thereby releasing drugs [76].

Epilepsy nanotherapeutic systems can utilize more than one type of stimulus-responsive method. Multi-stimuli-responsive nanomaterials, capable of addressing various aspects of the pathophysiology of epilepsy, hold promise as excellent carriers for ASMs.

## Conclusions

Nanotechnology has made significant progress in epilepsy treatment. Nanocarrier systems facilitate the passage of ASMs through the BBB, enhance the concentration of antiseizure medications in the brain, and target epileptic foci more precisely, thereby reducing adverse reactions. Nanosystems hold broad application prospects for the responsive delivery of ASMs. Epilepsy, with its unique properties, differs from other neurological disorders. Exploring how to utilize the specific pathophysiological mechanisms of epilepsy for more intelligent drug delivery is a topic worth investigating. How to achieve drug enrichment at epileptic discharge foci and rapidly and effectively suppress seizures during an epileptic event remains a research focus, likely requiring extensive interdisciplinary and cross-sectoral collaboration. In summary, it is hoped that nanotechnology can soon be applied in the clinical treatment of epilepsy.

## Data Availability

Availability of data and materials is not applicable in this study.

## Abbreviations

AMT: Adsorptive-mediated transcytosis
ASMs: Antiseizure medications
BBB: Blood-brain barrier
CaP: Calcium phosphate
CPs: Conducting polymers
CYP3A4: Cytochrome P450 3A4
D-T7: D-form T7
DON: 6-Diazo-5-oxo-L-norleucine
DRE: Drug-resistant epilepsy
DSPE: 1,2-Distearoyl-sn-glycero-3-phosphoethanolamine
Fc: Ferrocene
GLUT1: Glucose transporter 1
KCZ: Ketoconazole
LRP1: Low-density lipoprotein receptor-related protein 1
LAT1: L-type amino acid transporter 1
LTG: Lamotrigine
NaSS: Sodium 4-vinylbenzene sulfonate
PDA: Polydopamine
PEO-PPO-PEO: Polyethylene oxide-polypropylene oxide-polyethylene oxide
PF127: Poloxamer 407
P-gp: P-glycoprotein
PHT: Phenytoin
PLGA: Poly lactic-co-glycolic acid
PPY: Polypyrrole
PXR: Pregnane X Receptor
ROS: Reactive oxygen species
SPIONs: Superparamagnetic iron oxide nanoparticles
TAT: Trans-activator of transcription
TD: Tryptophan derivate
TfR: Transferrin receptor
TPGS: D-α-tocopheryl polyethylene glycol succinate
USPIO: Ultrasmall superparamagnetic iron oxide
